# Quality of life in the Iranian Blind War Survivors in 2007: a cross-sectional study

**DOI:** 10.1186/1472-698X-10-21

**Published:** 2010-08-21

**Authors:** Reza Amini, Hamid Haghani, Mehdi Masoumi

**Affiliations:** 1 Janbazan Medical and Engineering Research Center, Tehran, Iran; 2 Iran Medical Science School, Tehran, Iran

## Abstract

**Background:**

Quality of Life measurements are necessary tools for effectively evaluating health services. In the population of patients afflicted with war-related blindness in Iran, such measurements have yet to be documented and utilized. "The design and implementation of this study involved the determination of a baseline score for QOL in a population of Iranian blinded in the Iraq-Iran war in order to facilitate the design of interventions intended to improve the population's QOL."

**Methods:**

This was a cross-sectional study of a representative population of 250 war victims blind in both eyes at a 14-day recreational conference.

**Results:**

Participants had a mean age of 43.20(SD8.34) and their composition was 96.5% male and 3.5% female with a mean SF-36 QOL score of 59.20(SD22.80). An increasing level of education among the participants correlated with a higher QOL score (p = 0.006). The QOL also has a significant correlation to number of injuries (p < 0.0001). High systolic and diastolic blood pressure, hearing loss, and tinnitus had negative individual correlations to QOL (p = 0.016, 0.016, 0.005, p < 0.0001). The male sexual disorders of erectile dysfunction and premature ejaculation both had significant correlations to QOL (p = 0.026, p < 0.0001). Hypercholesterolemia showed significant correlation to QOL (p = 0.021).

**Conclusions:**

As blind war survivors' age, they will present with a greater set of burdens despite their relatively better QOL in the physical component scale when compared with lower limb amputees. Risk factors of cardiovascular attack such as high blood pressure and hypercholesterolemia were present and need future interventions.

**Key words:**

Quality of life, blindness, SF36, health

## Background

Armed conflict has been established as a major trigger for multiple physical, mental, and social injuries in all environments. Visual impairment and blindness are common afflictions present in one or both eyes as a result of such conflicts. As one of the longest modern wars, the Iran-Iraq War is no exception to this phenomenon and has produced many survivors with these injuries [1].

Health-related quality of life (HQOL) is a complex measurement that aggregates a range of variables that, as a whole, relate to individual's ability to lead a normal lifestyle. Assessing the relation between these vexatious variables and quality of life can help establish and run a more efficient and effective health system [2].

Blindness has a direct effect on quality of life; though coping with this situation is significantly less difficult if the blindness started before adulthood. Such individuals are more able to adapt their lifestyles when compared with those who became blind as adults and are thus capable of returning to day-to-day life sooner [2]. As a result, blindness or visual impairment in adulthood also tends to breed greater physical and mental issues [3]. Since most Iranian war survivors lost their vision around the age of 18,[1] knowing the baseline quality of life can be extremely useful in determining the quality and efficacy of health services as well as tracking changes over time [4,5].

In addition to physical impairment, mental problems, especially depression, have considerable prevalence in blind survivors [1]. These mental problems have significant effects on quality of life that become more obvious when correlated with physical factors [6]. Nonetheless, the effects of physical impairments and disabilities on quality of life are absolutely critical, considering the compounding negative role the aging process contributes to these physical problems. This negative role decreases quality of life much more than blindness alone [7]. Blind war survivors with accompanying physical and mental injuries will have additional impacts on their quality of life [8].

Gender as a variable can also play a role in quality of life as shown in female American veterans who scored lower than male veterans [4,9]. With this knowledge, decision-makers should take genders differences into account when formulating policies.

Age is another significant variable in the overall measurement of quality of life. Though controversy surrounds the extent and means by which some of the mechanisms of the aging process affect quality of life, it is well-established that the general process does have a major negative influence on physical well-being. Many co-morbidities like hypertension, traumatic injury, alcohol-related illnesses, and arthritis increase in prevalence with age, contributing to the overall deterioration of quality of life [10,11].

In light of the deficiency of quality of life data for blind war survivors in the Martyrs and Janbazan (veterans) Foundation of Iran and considering the importance of these criteria, this study was designed not only to describe this population, but also to establish a baseline for the evaluating the effectiveness of relevant plans and services.

## Methods

This cross-sectional study was of a convenience sample of 248 out of 250 completely blind Iranian survivors of the Iran-Iraq War who had agreed out of an initial group of 500 invitees to attend an educational and recreational overnight event in Mashhad, Iran. There was no exclusion criterion and all but two event attendees consented to taking part in the study. Ethical considerations were taken into account when designing and executing this study and the ethics board of the Janbazan (veterans) Medical and Engineering Research Center approved the project. The distribution of the participants was reflective of the Iranian blind veteran population based on demographic data previously gathered through a questionnaire [1]. The needs assessment questionnaire had previously been pilot tested for reliability and validity (additional file 1). The quality of life assessment used for both the victims and their spouses was done using the SF-36 Health Survey (additional files 2 and 3). Two trained surveyors assessed the victims and an additional three surveyors assessed the victim's spouses. The mean survey completion time was 10(2.5) minutes. A qualified internist also recorded various measurements of physical health using a provided data sheet (additional file 4). Blood pressure was evaluated in both the supine and sitting-position with a 10-minute rest between the measurements. Data from scheduled laboratory tests which have been done before the overnight event was also used.

Hypercholesterolemia was considered to be any measurement above 126, high systolic blood pressure any measurement above 140 mmHg, and high diastolic blood pressure any measurement above 90 mmHg [12].

In the field, data cleaning was done before two trained operators entered data into an SPSS file. The data entry error was 0.5%, which was subsequently resolved by file and questionnaires review. T-test, ANOVA and Pearson were performed as analysis tests with a resulting significant measurement of 0.05. All the means presented accompanied by standard deviations as Mean(SD).

## Results

**Figure 1:**
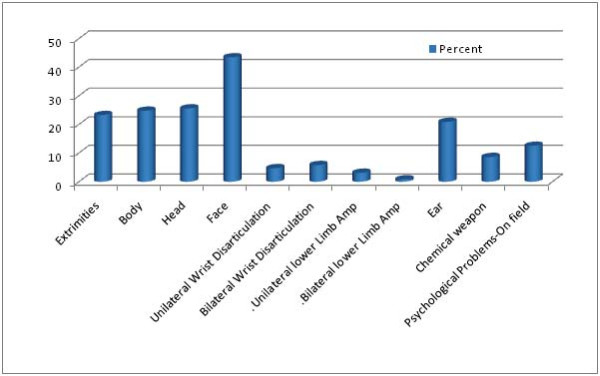
**Co-morbid Injuries in Blind War Survivors**.

The great majority of the sample in this study was male (96.5%) with a mean age of 43.20(8.34). The employment rate (19.10%) was almost one-fourth the unemployment rate (80.90%). Most were married (94.4%) and no divorce was reported. Education levels were reported to be less than a high school diploma (31.1%), high school diploma (28.3%), Associate's Degree (3.6%), Bachelor's Degree (20.7%), Master's Degree (13.5%), and PhD (2.8%). The length of blindness varied according to the time of injury and ranged between 1 and 29 years, however 79.2% of the sample had been blind length for 20 years at the time of study. Explosive trauma was reported as the main cause of injury. The number and different types of physical injuries accompanied by blindness were also reported (Table 1, Figure 1).

**Table 1:** Age and QOL in blind survivors

Age	n	QOL score Mean(SD)	
		
		PCS†	MCS‡
≤29	16	68.75(20.57)	62.13(22.03)

30-39	51	61.04(24.34)	58.18(25.55)

40-49	146	61.43(22.99)	59.89(22.82)

≥50	35	46.94(21.26)	50.29(19.83)

Total	248	59.78(23.43)	58.34(23.08)

^† ^The differences of scores between groups were significant (p = 0.003, f = 4.786) (ANOVA)

^‡ ^The differences of scores between groups were not significant (p = 0.157, f = 1.752) (ANOVA)

Quality of life in this group had an overall mean score of 59.2(22.8), a mean physical component scale (PCS) of 59.7(23.4) and for mental component scale (MCS), 58.1(23.1). The overall score and PCS had a significant correlation to aging (p = 0.009, 0.003), though the MCS did not (p = 0.157). Differences between those below and above the age 50 in overall score (p = 0.001) and also both the MCS (p = 0.015) and PCS (p < 0.0001) were significant.

While employment and QOL had no significant correlation (p = 0.241), there was indeed a significant correlation between the level of education and the overall QOL score, especially at the high school diploma and graduate levels (p = 0.006) (Table 2). MCS also had a significant correlation to education (p = 0.001, f = 5.788). Noticeable changes in overall QOL scores occur at the Bachelor's Degree level of education, wherein significant differences were perceivable between the scores of those having an Associate's Degree or less compared with those having a Master's Degree or higher.

**Table 2:** Education levels and QOL in blind survivors*

Education level	QOL score Mean(SD)	n
Less than a High School Diploma	57.20(23.37)	76

High School Diploma or 2 year degree	54.00(23.26)	79

Bachelors Degree	64.97(20.61)	48

Masters Degree or higher	66.43(18.27)	40

Total	59.21(22.47)	243

* The differences of total score between groups were significant (p = 0.006, f = 4.208) (ANOVA).

Physically injured survivors scored lower as a group, especially in the PCS (p = 0.004), though the MCS was also significantly lower (p =0.018) (Table 3).

**Table 3:** Physical injuries accompanied by blindness and QOL in war survivors*

Number of injuries	QOL score Mean(SD)	n
0	67.32(22.20)	47

1	64.19(19.90)	73

2	54.06(23.31)	52

3	54.10(25.15)	29

4	54.07(24.07)	29

5-6	49.94(19.76)	18

*Total*	59.26(22.84)	248

* The differences of total score between groups were significant (p = 0.003, f = 3.788) (ANOVA).

Systolic hypertension, diastolic hypertension, tinnitus, and hearing loss showed significant correlation to total QOL score, PCS, and MCS. Erectile dysfunction, premature ejaculation, and hypercholesterolemia showed significant relation to QOL total score (Table 4).

**Table 4:** Mean scores of QOL in terms of secondary factors affecting QOL in blind survivors

Secondary Factor		QOL Score Mean(SD)					
		
		Total	*P *value*	MCS	*P *value*	PCS	*P *value*
*Systolic HTN*	Yes	43.50(22.68)	0.018	44.00(23.00)	0.030	43.75(20.77)	0.016
						
	No	59.49(22.41)		58.98(23.13)		60.50(23.43)	

*Diastolic HTN*	Yes	49.82(27.20)	0.016	49.70(27.46)	0.022	51.14(26.78)	0.025
						
	No	60.00(21.83)		59.54(22.27)		60.92(22.80)	

*Tinnitus*	Yes	56.67(22.28)	0.005	56.55(22.92)	0.023	57.85(22.67)	0.005
						
	No	65.34(22.53)		63.74(23.23)		66.53(22.98)	

*Hearing loss*	Yes	55.37(21.87)	0.000	55.09(22.24)	0.000	56.80(22.53)	0.000
						
	No	67.07(20.63)		65.91(21.88)		67.83(21.20)	

*Premature ejaculation*	Yes	59.13(22.28)	0.000	59.02(22.90)	0.001	60.07(22.83)	0.000
						
	No	75.94(12.21)		71.63(13.24)		77.68(12.60)	

*Erectile dysfunction*	Yes	59.74(21.92)	0.026	59.34(22.51)	0.048	60.63(22.50)	0.021
						
	No	71.20(20.32)		69.75(19.21)		72.85(21.07)	

Hypercholesterolemia	Yes	56.06(22.31)	0.021	55.70(22.80)	0.081	57.54(23.59)	0.011
						
	No	65.54(19.44)		63.00(19.70)		68.47(20.75)	

						
*Abnormal HDL*	Yes	58.61(21.39)	0.031	57.97(21.70)	0.033	60.13(22.86)	0.030
						
	No	71.26(16.87)		70.93(21.98)		71.33(16.59)	

*t-test

Though the survivors' QOL scores had a significant correlation to their spouses' QOL scores (p < 0.0001, r = 0.1), more than 90% of the changes were related to other factors other than their spouses' QOL.

## Discussion

### Limitations

According to the relation between psychological problems and QOL, the lack of mental health data in this paper is a substantial limitation that should be considered in the future papers.

## Conclusions

One of the most important criteria in the evaluation of the effectiveness of health services and of patient health is QOL. Assessing QOL can inform decision makers of the variables that affect QOL most so they can adjust their policies accordingly.

In this sample population, the age distribution revealed that in the next decade, many of the blind survivors will begin to enter advanced age. During this period, their physical condition will deteriorate and they will develop special needs, markedly decreasing their QOL [13]. A comparison of the blind survivors samples below and above 50 year showed a marked decrease in all QOL scores after patients crossed the 50 year threshold. Thus, the portion of the sample population in the 40-49 age range demonstrates a pressing need for rehabilitation as they approach a critical point in their lives.

Hypertension and hypercholesterolemia have been established as synergistic risk factors in the development of cardiovascular diseases and cardiac arrest. By the measurement of these two factors, this study sought to show the correlation between blindness and cardiovascular conditions. As the population ages and becomes less physically active, the risk of developing hypertension or hypercholesterolemia becomes even greater.

This study's results reaffirmed previous findings drawn from a sample of blind individuals in 2003 [3].

Formal education, especially at the post-secondary levels, could be helpful to improve the QOL of blind individuals [1]. Those without a high school diploma would be at risk for a low QOL score. Since most of the sample was blinded by explosive trauma, this population also suffered other physical impairments and disabilities [1]. Multiple injuries and trauma had a significant effect on QOL, with the number of injuries negatively correlating to QOL. Highly specialized design and methods considering their physical limitations could be helpful to promote their educational level.

Sexual dysfunction should be resolved through a multifaceted approach with drug therapy and education.

Population growth and lower mortality rates have increased both the proportion and number of elderly individuals around the world, multiplying the general rate of physical disabilities such as visual impairment and blindness in the population. By 2050, the average global life expectancy will increase by about 10 years--an increase that will correlate with a tripling of the over 65 population in developing nations [14]. Blindness due to any cause can make lower QOL in an afflicted population, and this study's findings suggest which areas policymakers should focus on in order to rectify this.

Most geriatric quality of life studies have shifted their outlook toward advanced age from pessimism to optimism. This new attitude stipulates that old age is simply another stage of the human lifespan during which people attain new roles and responsibilities. In this period, people find themselves in new situations with new demands, thus demonstrating the need to reform rehabilitation methods for this population [15].

## List of Abbreviations

QOL: Quality Of Life; ADL: Activity Daily Living; MCS: Mental Component Scale; PCS: Physical Component Scale; BLLA: Bilateral Lower limb Amputation

## Competing interests

The authors declare that they have no competing interests.

## Authors' contributions

RA carried out the design, data acquisition, drafting and finalizing the manuscript and participated in statistical analysis. HH carried out statistical analysis, and participated in designing the study and also drafting and finalizing the manuscript. MM participated in the design, coordination and also drafting the manuscript. All authors read and approved the final manuscript.

## Pre-publication history

The pre-publication history for this paper can be accessed here:

http://www.biomedcentral.com/1472-698X/10/21/prepub

## Supplementary Material

Additional file 1 **Needs Assessment**. This file contains some demographic data and some questions about the demands of war survivors with blindness. The questionnaire is in Persian language. Click here for file

Additional file 2 **Blind war survivors' health related quality of life**. The file contains standard SF36 Persian version Click here for file

Additional file 3 **Health related quality of life in the blind war survivors' spouse**. The file contains standard SF36 Persian version Click here for file

Additional file 4 **General Physical Exam**. The file contains history and physical exam of the blind war survivors. Click here for file
