# Supplementary material for: Quality of life in the Iranian Blind War Survivors in 2007: a cross-sectional study
Source: BMC Int Health Hum Rights. 2010 Aug 21;10:21. doi: 10.1186/1472-698X-10-21 (PMC2936407; doi:10.1186/1472-698X-10-21)
Supplement: Additional file 1 — Needs Assessment. This file contains some demographic data and some questions about the demands of war survivors with blindness. The questionnaire is in Persian language. [file 1472-698X-10-21-S1.DOCX]

**1- محل سکونت - 1-1 استان: . . . . . . . . . . . . . . . . . . . . . . . . . . . . . . . . . . . . . . . . . . . 2-1 شهرستان : . . . . . . . . . . . . . . . . . . . . . . . . . . . . . . . . . . . . . . . . . . . . . . . . . .**

**2- جنس: مرد^1^ ○ زن^2^○ 3- سال تولد**: ⬜⬜  **4- سال مجروحيت:** ⬜⬜ **5- سال نابينايی:**⬜⬜ **6-تخليه چشم:** چپ⬜ راست⬜

**7- مجروحيت همراه:** 1-7 ترکش به اندام⭘ 2-7 ترکش به بدن⭘ 3-7 ترکش به سر ⭘ 4-7 ترکش به صورت⭘ 5-7 دندان ها⭘

6-7 قطع يک مچ⭘ 7-7 قطع دو مچ⭘ 8-7 قطع يک پا⭘ 9-7 قطع دوپا⭘ 10-7 گوش⭘

11-7 شيميايی⭘ 12-7 اعصاب و روان⭘ 13-7 ساير: . . . . . . . . . . . . . . . . . . . . . . . . . . . . . . . . . . . .

**تحصيلات: 8- جانباز , قبل از نابينايی 9- جانباز , پس از نابينايی**

زيرديپلم^1^ ديپلم^2^ فوق ديپلم^3^ ليسانس^4^ فوق ليسانس^5^ دکترا^6^

زيرديپلم^1^ ديپلم^2^ فوق ديپلم^3^ ليسانس^4^ فوق ليسانس^5^ دکترا^6^

○ ○ ○ ○ ○ ○

○ ○ ○ ○ ○ ○

**10- طول مدت تحصيل در آخرين مقطع:** ⬜⬜ **سال**

**11- همسر 12- فرزند 1**

زيرديپلم^1^ ديپلم^2^ فوق ديپلم^3^ ليسانس^4^ فوق ليسانس^5^ دکترا^6^

ابتدايی^1^ راهنمايی^2^ دبيرستان^3^ ديپلم^4^ ليسانس و بالاتر^5^

○ ○ ○ ○ ○ ○

○ ○ ○ ○ ○

**13- فرزند2 14- فرزند 3**

ابتدايی^1^ راهنمايی^2^ دبيرستان^3^ ديپلم^4^ ليسانس و بالاتر^5^

ابتدايی^1^ راهنمايی^2^ دبيرستان^3^ ديپلم^4^ ليسانس و بالاتر^5^

○ ○ ○ ○ ○

○ ○ ○ ○ ○

**15- وضعيت تأهل قبل از نابينايی :** متأهل^1^○ مجرد^2^○  **16- وضعيت تأهل پس از نابينايی:** متأهل^1^○ مجرد^2^○

**17-**  **سال ازدواج:**⬜⬜ **18- تعداد افراد تحت تکفل**:⬜⬜

**19- محل سکونت-** شهر^1^○ روستا^2^ ○  **20- مالکيت :** شخصی^1^ ○ استيجاری^2^ ○

**21- نوع منزل :**  خانه^1^ ○ آپارتمان^2^ ○ 🡨 طبقه ⬜⬜ - آسانسور: دارد^1^○ ندارد^2^○

**شغل – 22- شغل فعلي :** حالت اشتغال^1^○ بی کار^2^○ شاغل^3^○ 🡨 نوع شغل درحال حاضر: . . . . . . . . . . . . . . . . . . . .

***1-22 شغل قبل از نابينايی*** *: بی کار⭘ دانش آموز⭘ دانشجو⭘ کارمند⭘ کارگر⭘ نظامی⭘ ساير: . . . . . . . . . . . . . . . . . . .*

***2-22 شغل پس از نابينايی :*** بی کار⭘ دانش آموز⭘ دانشجو⭘ کارمند⭘ کارگر⭘ نظامی⭘ ساير: . . . . . . . . . . . . . . .

**درصورت شاغل بودن🡨 23- فاصله محل کار از منزل:** ⬜⬜⬜ کيلومتر

**24- نحوه تردد به محل کار: -** پياده ⭘ اتومبيل شخصی⭘ تاکسی⭘ اتوبوس⭘ آژانس⭘ سرويس⭘

1-24- با همراه ○ به تنهايی○ 1-1-24 اگر به تنهايی 🡨 با کمک مردم○ بدون کمک مردم○

**25- آيا ديگر اعضای تحت تکفل شما نيز شاغل هستند؟** خير^0^○ بلی^1^ ○ 🡨 1-25- همسر ⭘ 2-25- فرزند⭘ 3-25- والدين⭘

**26- اگر ديگر اعضای خانواده نيز شاغل هستند, ميزان درآمد ماهانه خانواده:**

بيش از يك ميليون تومان^1^ ○ پانصدهزار تا يك‌ميليون تومان^2^⭘ چهارصد تا پانصدهزار تومان^3^○ سيصد تا چهارصدهزارتومان^4^○ دويست تا سيصد هزارتومان^5^○ يکصد تا دويست هزار تومان^6^ ○ کمتر از يکصد هزار تومان^7^○

**27-  *ميزان درآمد ماهانه شما:*** بيش از يك ميليون تومان^1^ ○ پانصدهزار تا يك‌ميليون تومان^2^⭘ چهارصد تا پانصدهزار تومان^3^○ سيصد تا چهارصدهزارتومان^4^○ دويست تا سيصد هزارتومان^5^○ يکصد تا دويست هزار تومان^6^ ○ کمتر از يکصد هزار تومان^7^○

**روابط فردی و اجتماعی:**

**28- وجود معلوليت در ديگر افراد خانواده:**  1-28 همسر ⭘ 2-28 فرزند ⭘ 3-28 پدر ⭘ 4-28 مادر ⭘

**29- تا چه ميزان, در انجام امور منزل مشارکت می کنيد؟** بسيار زياد^1^○ زياد^2^○ کم^3^○ بسيار کم^4^○ امتناع از پاسخ^5^○

1-29 چه فعاليت هايی را انجام می دهيد؟ 1-1-29خريد ⭘ 2-1-29 آشپزی⭘ 3-1-29 نظافت⭘ 4-1-29 ظرف شستن⭘

5-1-29 تعمير لوازم منزل⭘ 6-1-29 نظارت تحصيلی فرزندان⭘ 7-1-29 ساير: . . . . . . . . .

**30- درصورتی که پاسخ سؤال 29 ,کم و يا بسيارکم است ؛ عامل پايين بودن مشارکت:**

1-30 عدم اطمينان خانواده ⭘ 2-30 پايين بودن اعتماد به نفس من موجب عدم تمايل من می شود⭘ 3-30 محدوديت زمان حضور در منزل⭘

4-30 ناتوانی در انجام امور⭘ 5-30 نامناسب بودن محيط خانه ⭘ 6-30 محبت بيش از اندازه خانواده⭘

**31- اعضای خانواده تا چه ميزان, از شما در انجام امور منزل کمک می خواهند؟**

بسيار زياد^1^○ زياد^2^○ کم^3^○ بسيار کم^4^○ امتناع از پاسخ^5^○

**32- اعضای خانواده برای مشورت در تصميم گيری ها, تا چه ميزان از شما نظر خواهی می کنند؟**

در تمام امور^1^○ در هيچ يک از امور^2^○ در امور خاص^3^○ ( مثل : خريد، تهيه لباس، تحصيل فرزندان، هزينه ها و بودجه زندگي و . . . )

**33- تا چه ميزان, در گفتگوهای خانواده شرکت می کنيد؟**

بسيار زياد^1^○ زياد^2^○ کم^3^○ بسيار کم^4^○ امتناع از پاسخ^5^○

**34- تا چه ميزان, در گفتگوهای همکاران و يا دوستان خود شرکت می کنيد؟**

بسيار زياد^1^○ زياد^2^○ کم^3^○ بسيار کم^4^○ امتناع از پاسخ^5^○

**35- تا چه ميزان, برای خريد روزانه از خانه خارج می شويد؟**

بسيار زياد^1^○ زياد^2^○ کم^3^○ بسيار کم^4^○ امتناع از پاسخ^5^○ اصلا^6^○

درصورتی که پاسخ ؛ کم, بسيارکم و يا اصلا است 🡨 1-35 علت: مشکلات جسمی⭘ تردد⭘ محدوديت زمان⭘ ساير . . . .

**36- معمولا برای چه مدتی از آينده خود برنامه ريزی می کنيد؟** سالانه⭘ ماهانه⭘ هفتگی⭘ روزانه⭘ بدون برنامه⭘

**37- چند ساعت را در هفته به تفريح اختصاص می دهيد؟**بيش از 6 ساعت^1^ ○ 4 تا 6 ساعت^2^○ 2 تا 4 ساعت^3^ ○ کمتر از 2 ساعت^4^ ○

**38- از اين زمان چه ميزان را با خانواده همراه هستيد؟** تمام ساعات^1^○ نيمی از ساعات^2^ ○ زمان مختصری^3^ ○ به تنهايی تفريح می کنم^4^ ○

**39- به چه ميزان از مکان های تفريحی که در شهرستان شما وجوددارد , استفاده می کنيد؟**

بسيار زياد^1^○ زياد^2^○ کم^3^○ بسيار کم^4^○ امتناع از پاسخ^5^○

**40- آيا به همراه خانواده, به مکان های تفريحی که برای شما خيلی قابل استفاده نيستند (مثل سينما), می رويد؟**

بسيار زياد^1^○ زياد^2^○ کم^3^○ بسيار کم^4^○ امتناع از پاسخ^5^○

**41- از چه وسيله ای برای کسب اطلاعات بيشتراستفاده می کنيد؟**

1-41 روزنامه نابينايان ⭘ 2-41 روزنامه با کمک ديگران ⭘ 3-41 راديو ⭘ 4-41 تلويزيون ⭘ 5-41 نشريات ⭘ 6-41کامپيوتر و اينترنت ⭘ 7-41 تلفن گويا ⭘ 8-41 هيچکدام ⭘ 9-41 علت: . . . . . . . . . . . . . . . . . . . . . . . . . . . . . . . . . . . . . . . . . . . . . . . . . . . . . . . . . . . . . . . . . . . . . . .

**42- آيا نابينايی, در انجام وظايف پدری شما تأثيری داشته است؟**

بسيار زياد^1^○ زياد^2^○ کم^3^○ بسيار کم^4^○ امتناع از پاسخ^5^○

**43- فکر می کنيد که ميزان رضايت خانواده از نقش شما در خانواده, چگونه است؟**

بسيار زياد^1^○ زياد^2^○ کم^3^○ بسيار کم^4^○ امتناع از پاسخ^5^○

**44- ميزان رضايت شما, از رفتار همسرتان با شما چگونه است؟**

بسيار زياد^1^○ زياد^2^○ کم^3^○ بسيار کم^4^○ امتناع از پاسخ^5^○

**45- ميزان رضايت شما, از رفتار فرزندان با شما چگونه است؟**

بسيار زياد^1^○ زياد^2^○ کم^3^○ بسيار کم^4^○ امتناع از پاسخ^5^○

**46- ميزان رضايت شما, از رفتار دوستان و همکاران با شما چگونه است؟**

بسيار زياد^1^○ زياد^2^○ کم^3^○ بسيار کم^4^○ امتناع از پاسخ^5^○

**47- فکر می کنيد که ميزان رضايت همسر شما از رفتار شما با او چگونه است؟**

بسيار زياد^1^○ زياد^2^○ کم^3^○ بسيار کم^4^○ امتناع از پاسخ^5^○

**48- فکر می کنيد که ميزان رضايت فرزندان از رفتار شما با ايشان چگونه است؟**

بسيار زياد^1^○ زياد^2^○ کم^3^○ بسيار کم^4^○ امتناع از پاسخ^5^○

**49- فکر می کنيد که ميزان رضايت همکاران و دوستان, از رفتار شما با آن ها چگونه است؟**

بسيار زياد^1^○ زياد^2^○ کم^3^○ بسيار کم^4^○ امتناع از پاسخ^5^○

**50- آيا تاکنون با کسی که ناآشنا بوده است, از طريق تلفن, مکاتبه, اينترنت و. . . ارتباط برقرار کرده ايد؟** خير○ بلی ○

**-در صورت بلی 🡨 1-50 آيا پس از اين که متوجه نابينايی شما شد, نحوه برخورد وی تغييری کرد؟** خير○ بلی ○ 🡫

1-1-50ترحم ⭘ 2-1-50 تشويق ⭘ 3-1-50 ناديده گرفتن(با فرد ديگری ارتباط برقرار کرده است) ⭘ 4-1-50 عدم درک شرايط يک فرد نابينا ⭘ 5-1-50 قطع ارتباط ⭘ 6-1-50 رفتار کودکانه (مانند يک کودک با شما رفتار کرده باشد) ⭘ 7-1-50دوستانه ⭘ 8-1-50تمسخر ⭘ 9-1-50 عذرخواهی ⭘

**51- آيا عضو انجمن, شورا و يا تشکل خاصی (**مثل؛ شورای شهر, شورای مسجد, NGO و . . . **) هستيد ؟** خير^0^○ بلی^1^○ امتناع از پاسخ^3^○

**52- آيا تا کنون در انتخابات شرکت کرده ايد ؟**  بلی^1^○ 🡨 کشوری 🞎 استانی 🞎 شهرستان 🞎 روستا🞎

خير^0^○

**53- آيا تا کنون در انتخابات خاصی کانديدا شده ايد؟** خير^0^○ بلی^1^○ 🡨 کشوری 🞎 استانی 🞎 شهرستان 🞎 روستا 🞎

**54- آيا در روابط اجتماعی بين خود و ديگران احساس تبعيض کرده ايد؟** خير^0^○ بلی^1^○

**55- ميزان رضايت شما از حمايت بنياد, در فعاليت های اجتماعی شما چگونه است؟** بسيار زياد^1^○ زياد^2^○ کم^3^○ بسيار کم^4^○ امتناع از پاسخ^5^○

**56- ميزان رضايت شما از حمايت ادارات استان (**غيراز بنياد**), در فعاليت های اجتماعی شما چگونه است؟**

بسيار زياد^1^○ زياد^2^○ کم^3^○ بسيار کم^4^○ امتناع از پاسخ^5^○

**57- ميزان رضايت شما از حمايت خانواده, در فعاليت های اجتماعی شما چگونه است؟**

بسيار زياد^1^○ زياد^2^○ کم^3^○ بسيار کم^4^○ امتناع از پاسخ^5^○

**درصورتی که تحصيلات دانشگاهی داشته ايد و يا درحال حاضر دانشجو هستيد :**

**58 - همخوانی رشته تحصيلی شما با توانايی های شما چگونه بود/ است؟ (**و يا اگر در حال تحصيل هستيد**)**

بسيار زياد^1^○ زياد^2^○ کم^3^○ بسيار کم^4^○ امتناع از پاسخ^5^○

**59- ميزان رضايت شما از روش های ارائه مطالب توسط اساتيد چگونه بود/است؟**

بسيار زياد^1^○ زياد^2^○ کم^3^○ بسيار کم^4^○ امتناع از پاسخ^5^○

**60- ميزان آشنايی دانشجويان با موضوع نابينايی چگونه بود/است؟**

بسيار زياد^1^○ زياد^2^○ کم^3^○ بسيار کم^4^○ امتناع از پاسخ^5^○

**61- ميزان آشنايی اساتيد با موضوع نابينايی چگونه بود/است؟**

بسيار زياد^1^○ زياد^2^○ کم^3^○ بسيار کم^4^○ امتناع از پاسخ^5^○

**62- ميزان رضايت شما از مراجع و کتب چگونه بود/است؟** بسيار زياد^1^○ زياد^2^○ کم^3^○ بسيار کم^4^○ امتناع از پاسخ^5^○

**63- ميزان رضايت شما از امکانات آموزشی (**مثل کتابخانه, سايت و . . . **) چگونه بود/است؟**

بسيار زياد^1^○ زياد^2^○ کم^3^○ بسيار کم^4^○ امتناع از پاسخ^5^○

**64- ميزان رضايت شما از روش های ارزشيابی و امتحانات چگونه بود/است؟**

بسيار زياد^1^○ زياد^2^○ کم^3^○ بسيار کم^4^○ امتناع از پاسخ^5^○

**شغل(در صورت شاغل بودن جانباز سؤال شود)**

**65- برای انجام وظايف شغلی خود از کسی کمک می خواهيد؟**

بسيار زياد^1^○ زياد^2^○ کم^3^○ بسيار کم^4^○ امتناع از پاسخ^5^○

**66- چگونه از قابليت های شما, در شغل فعلی استفاده می شود؟**

بسيار زياد^1^○ زياد^2^○ کم^3^○ بسيار کم^4^○ امتناع از پاسخ^5^○

**67- ميزان علاقه شما به شغل فعلی خود چقدر است؟**

بسيار زياد^1^○ زياد^2^○ کم^3^○ بسيار کم^4^○ امتناع از پاسخ^5^○

**68- آيا تا کنون در محيط کار به سبب انجام وظايف و يا جابجايی دچار حوادث جدی شده ايد؟** خير^0^○ بلی^1^○ 🡫

1-68 ذکر نماييد؛ . . . . . . . . . . . . . . . . . . . . . . . . . . . . . . . . . . . . . . . . . . . . . . . . . . . . . . . . . . . . . . . . . . . . . . . . . . . . . . . . . . . . . . . . . . . . . . . . .

**69- اگر همکاران شما وسيله ای را جابجا کنند :**

به من اطلاع می دهند○^1^ پس از اين که محل آن را از آن ها بپرسم به من کمک می کنند○^2^ اصلا برای پيدا کردن آن به من کمکی نمی کنند○^3^

**70- آيا شغل فعلی شما, نياز به توانايی خاصی دارد که شما قادر به انجام آن نباشيد؟** خير^0^○ بلی^1^○ 🡫

1-70 ذکر نماييد؛ . . . . . . . . . . . . . . . . . . . . . . . . . . . . . . . . . . . . . . . . . . . . . . . . . . . . . . . . . . . . . . . . . . . . . . . . . . . . . . . . . . . . . . . . . . . . . . . . . .

**71- آيا بين شما و ديگر همکارانتان که پست مشابه شما دارند, تبعيض قايل می شوند؟**

هميشه^1^○ اغلب^2^ ○ گاهی ^3^○ هرگز^4^○

**72- آيا تسهيلات ويژه ای برای شما در نظر گرفته شده است؟** خير^0^○ بلی^1^○ 🡫

1-72 ذکر نماييد؛ . . . . . . . . . . . . . . . . . . . . . . . . . . . . . . . . . . . . . . . . . . . . . . . . . . . . . . . . . . . . . . . . . . . . . . . .

**73- آيا از قوانين کار در خصوص نابينايان, اطلاع داريد؟**

کاملا اطلاع دارم^1^○ فقط در حيطه شغل خودم اطلاع دارم^2^○ تا حدودی اطلاع دارم^3^○ هيچ اطلاعی ندارم^4^○

**74- همخوانی شغل شما با توانايی‌هايتان چگونه است؟**

بسيار زياد^1^○ زياد^2^○ کم^3^○ بسيار کم^4^○ امتناع از پاسخ^5^○

**75- آيا برای بهبود کيفيت شغلی خود نياز به آموزش خاصی داريد؟** خير^0^○ بلی^1^○ 🡫

1-75 ذکر نماييد؛ . . . . . . . . . . . . . . . . . . . . . . . . . . . . . . . . . . . . . . . . . . . . . . . . . . . . . . . . . . . . . . . . . . . . . . . . . . . . . . . . . . . . . . . . . . . . . . . .

**76- در مجموع ميزان رضايت شما از شغلتان چگونه است؟**

بسيار زياد^1^○ زياد^2^○ کم^3^○ بسيار کم^4^○ امتناع از پاسخ^5^○

**بهداشت و درمان**

**77 - به طور متوسط, چند ساعت در شب می خوابيد؟** ⬜⬜  **78- به طور متوسط, چندساعت در روز می خوابيد؟** ⬜⬜

**79- به طور متوسط, شب ها از زمانی که به بستر می رويد تا زمانی که به خواب برويد چقدر طول می کشد؟**

بيش از يک ساعت○^1^ در حدود نيم ساعت○^2^ کمتر از يک ربع ساعت○^3^

**80- آيا تا کنون اتفاق افتاده است که انرژی و توان لازم را برای انجام وظايف خود نداشته باشيد؟** خير^0^○ بلی^1^○ 🡫

1-80 - هر روز○^1^ سه بار در هفته○^2^ يک بار در هفته○^3^ گاهی○^4^

**81- آيا رژيم غذايی خاصی داريد؟** خير^0^○ بلی^1^○

**82- آيا به خدمات درمانی خاصی (**مثل ويزيت های دوره ای,مراقبت های پرستاری و . . . **) نيازمند هستيد؟**

هميشه ○^1^ اغلب○ ^2^ گاهی○^3^ اصلا○^4^

**83- اگر به خدمت خاصی نيازمند هستيد نوع آن را ذکر کنيد**: . . . . . . . . . . . . . . . . . . . . . . . . . . . . . . . . . . . . . . . . . . . . . . . . . . . . . . . . . . . . . . . . .

. . . . . . . . . . . . . . . . . . . . . . . . . . . . . . . . . . . . . . . . . . . . . . . . . . . . . . . . . . . . . . . . . . . . . . . . . . . . . . . . . . . . . . . . . . . . . . . . . . . . . . . . . . . . . . . . . . .

**84- آيا عوارض جسمی ناشی از نابينايی را می شناسيد؟** خير^0^○ بلی^1^○ 🡫

1-84 چند مورد مهم را ذکر نماييد؛ . . . . . . . . . . . . . . . . . . . . . . . . . . . . . . . . . . . . . . . . . . . . . . . . . . . . . . . . . . . . . . . . . . . . . . . . . . . . . . . . . . . . .

**85- آيا به سبب مشكلات جسمي از كار غايب شده‌ايد؟** خير^0^○ بلی^1^○ 🡫

1-85 براي چه مدت؟ ⬜⬜ / ماه

**86- آيا از عوارض ناشی از نابينايی رنج می بريد؟** خير^0^○ بلی^1^○

1-86 چند مورد مهم را ذکر نماييد؛ . . . . . . . . . . . . . . . . . . . . . . . . . . . . . . . . . . . . . . . . . . . . . . . . . . . . . . . . . . . . . . . . . . . . . . . . . . . . . . . .

**87- آيا از وسيله مصرفی خاصی (**مثل پد چشمی و . . . **) استفاده می کنيد ؟** خير^0^○ بلی^1^○

**88- اگر پاسخ سؤال 87 بلی است, تهيه آن چگونه است؟** آسان و به موقع **○**^1^ با کمی تأخير و دشواری○ ^2^ بسيار دشوار و با تأخيرفراوان○^3^

**89- هزينه تهيه وسايل مصرفی را چگونه پرداخت می نماييد؟**

کل آن به عهده خودم می باشد○^1^ پس از پرداخت, از بنياد می گيرم○ ^2^ بنياد می پردازد/ تحويل می دهد○^3^

**90- به طور متوسط, در طول يك سال چندبار برای انجام امور درمانی به پزشک مراجعه می نماييد؟** ⬜⬜

**91- آخرين بار كه به پزشك مراجعه كرديد، چه زماني بود؟** ⬜⬜ /هفته

**92- آيا از داروی خاصی استفاده می کنيد؟** خير^0^○ بلی^1^○

**93- اگر پاسخ سؤال 92 بلی است , تهيه آن چگونه است؟**

ناياب است و تهيه آن بسيار دشوار است ○ از داروخانه های مشخصی تهيه می کنم○ بنياد تحويل می دهد○ در هر داروخانه ای موجود است○

**94- هزينه تهيه داروی خاص را چگونه پرداخت می نماييد؟**

کل آن به عهده خودم می باشد○ از بيمه عمومی و خودم○ بيمه تکميلی و بيمه عمومی○ پس از پرداخت, از بنياد می گيرم○ بنياد می پردازد○

**95- آيا از پروتز خاصی** (چشم، دست، پا و . . . ) **استفاده می کنيد؟** خير^0^○ بلی^1^○

**96- اگر پاسخ سؤال 97 بلی است, آن را چگونه تهيه می کنيد؟** از مراکز غير بنيادی تهيه می کنم○ بنياد تحويل می دهد○

**97- ميزان رضايت شما از نحوه دريافت آن چگونه است؟**

بسيار زياد^1^○ زياد^2^○ کم^3^○ بسيار کم^4^○ امتناع از پاسخ^5^○

**98- آشنايی کادر درمانی(**پزشک, پرستار, بهيار و . . . **) با مشکلات شما چگونه است؟**

بسيار زياد^1^○ زياد^2^○ کم^3^○ بسيار کم^4^○ امتناع از پاسخ^5^○

**99- اقدامات درمان لازم چگونه انجام می شود؟** کاملا به موقع○ با کمی تأخير○ پس از بروز مشکلات و عوارض شديد○

**100- آيا به سبب سوء درمان دچار عارضه خاصی شده ايد؟** خير^0^○ بلی^1^○🡫

1-100 چند مورد مهم را ذکر نماييد؛ . . . . . . . . . . . . . . . . . . . . . . . . . . . . . . . . . . . . . . . . . . . . . . . . . . . . . . . . . . . . . . . . . . . . . . . . . . . . . . . .

**101- نقش بنياد را تا کنون, در درمان خود چگونه ارزيابی می کنيد؟**

بسيار زياد^1^○ زياد^2^○ کم^3^○ بسيار کم^4^○ امتناع از پاسخ^5^○

**102- نقش بيمه تکميلی را تاکنون, در درمان خود چگونه ارزيابی می نماييد؟**

بسيار زياد^1^○ زياد^2^○ کم^3^○ بسيار کم^4^○ امتناع از پاسخ^5^○

**103- نقش بخش های دولتی (**مثل بيمارستان ها, درمانگاه ها و . . . **) را تاکنون, در درمان خود چگونه ارزيابی می کنيد؟**

بسيار زياد^1^○ زياد^2^○ کم^3^○ بسيار کم^4^○ امتناع از پاسخ^5^○

**توانبخشی**

**104- آيا وسايل توانبخشی ويژه نابينايان را می شناسيد؟** خير^0^○ بلی^1^○ 🡫

1-104 چند مورد مهم را ذکر نماييد؛ . . . . . . . . . . . . . . . . . . . . . . . . . . . . . . . . . . . . . . . . . . . . . . . . . . . . . . . . . . . . . . . . . . . . . . . . . . . . . . . . . . .

**105- آيا از وسيله توانبخشی ويژه نابينايان استفاده می کنيد؟** خير^0^○ بلی^1^○ 🡫

1---105 چند مورد مهم را ذکر نماييد؛ . . . . . . . . . . . . . . . . . . . . . . . . . . . . . . . . . . . . . . . . . . . . . . . . . . . . . . . . . . . . . . . . . . . . . . . . . . . . . . . . .

**106- برای استفاده از آن چگونه آموزش ديده ايد؟**

کارشناس بنياد ○ شرکت نمايندگی○ بهزيستی ○ به مرور خودم ياد گرفته ام○ از خانواده ام کمک گرفته ام○

**107- آيا علاوه بر نابينايی, ناتوانی ديگری هم داريد؟** خير^0^○ بلی^1^○ 🡫

1-107 ذکر نماييد؛ . . . . . . . . . . . . . . . . . . . . . . . . . . . . . . . . . . . . . . . . . . . . . . . . . . . . . . . . . . . . . . . . . . . . . . . . . . . . . . . . . . . . . . . . . . . . . . . . .

**108- آيا از وسيله کمکی خاصی برای ناتوانی های ديگر خود, غير از نابينايی، استفاده می کنيد ؟** خير^0^○ بلی^1^○ 🡫

1-108 ذکر نماييد؛ . . . . . . . . . . . . . . . . . . . . . . . . . . . . . . . . . . . . . . . . . . . . . . . . . . . . . . . . . . . . . . . . . . . . . . . . . . . . . . . . . . . . . . . . . . . . . . . . .

**109- آيا فعاليت ورزشی خاصی را انجام می دهيد؟** خير^0^○ بلی^1^○ 🡫

1-109 ذکر نماييد؛ . . . . . . . . . . . . . . . . . . . . . . . . . . . . . . . . . . . . . . . . . . . . . . . . . . . . . . . . . . . . . . . . . . . . . . . . . . . . . . . . . . . . . . . . . . . . . . . . . . .

**110- اگر پاسخ سؤال109 بلي است، هفته ای چندبار تمرين ورزشي می کنيد؟** ⬜⬜

**111- آيا در باشگاه و يا تيم خاصی عضويت داريد؟** خير^0^○ بلی^1^○

**تحرک , جابجايی و حمل و نقل**

**112- تا چه ميزان قادر هستيد فعاليت های روزمره و شخصی خود را (**بهداشت فردی, آراستگی ظاهری,استفاده از وسايل و . . .**) در منزل به تنهايی انجام دهيد؟**  تمام کارها○ بخش زيادی ○ کمی○ کليه کارهای شخصی ام را ديگران انجام می دهند○

**113- آيا محل وسايل را در منزل می دانيد؟** تمام وسايل ○ محل بيشتر وسايل را می دانم○

محل تعداد کمی از وسايل را می دانم○ برای هر وسيله ای بايد از ديگران کمک بگيرم○

**114- آيا اعضای خانواده هنگام جابجايی وسايل و لوازم منزل به شما اطلاع می دهند؟**

هميشه اطلاع می دهند ○ گاهی اوقات اطلاع می دهند ○ اغلب فراموش می کنند○ هيچ وقت به من اطلاع نمی دهند○

**115- در طی ماه گذشته, هنگام تردد در منزل, چندبار دچار حادثه جدی شده ايد؟**

بيش از 10 بار○ 5 تا 10 بار ○ کمتر از 5 بار○ هرگز○

**116- برای پيشگيری از حوادث مشابه چه کرده ايد؟**. . . . . . . . . . . . . . . . . . . . . . . . . . . . . . . . . . . . . . . . . . . . . . . . . . . . . . . . . . . . . . . . . . . . . . . . . .

**117- آيا برای تردد و جابجايی آموزش خاصی ديده ايد؟** خير^0^○ بلی^1^○

**118- آيا خانواده شما از روش های مناسب سازی محيط داخلی منزل آگاهی دارند؟** خير^0^○ بلی^1^○

**119- روزانه چند ساعت پياده روی می کنيد؟**⬜⬜ ساعت

**120- آيا هنگام خروج از منزل برای رسيدن به مقصد و يا برای سوار شدن به وسايل نقليه سمت و جهت حرکت را می دانيد؟**

اصلا کمک نمی خواهم ○ گاهی کمک می خواهم○ اغلب کمک می خواهم ○ هميشه کمک می خواهم○

**121- اگر برای اولين بار به يک محل وارد شويد, آيا می توانيد به تنهايی خارج شويد؟**

بدون کمک و راهنمايی ○ با کمی راهنمايی○ با کمک ○ حتما بايد کسی مرا خارج کند○

**122- تا چه ميزان برای جابجايی در شهر از وسايل نقليه عمومی (**تاکسی و اتوبوس**) استفاده می کنيد؟**

بسيار زياد^1^○ زياد^2^○ کم^3^○ بسيار کم^4^○ هرگز^5^○

1-122- اگر پاسخ كم، بسياركم و هرگز است، چرا؟ . . . . . . . . . . . . . . . . . . . . . . . . . . . . . . . . . . . . . . . . . . . . . . . . . . . . . . . . . . . . . . . . . . . . . . . . . . . . .

**123- آيا برای سوار و پياده شدن نياز به کمک داريد؟**

بدون کمک و راهنمايی ○ با کمی راهنمايی○ با کمک ○ حتما بايد کسی مرا پياده کند○

**124- آيا هنگام تردد در خيابان, مردم به شما کمک می کنند؟**

بسيار زياد^1^○ زياد^2^○ کم^3^○ بسيار کم^4^○ هرگز^5^○

**125- در زمان تردد و جابجايی , رفتارمردم با شما چگونه است؟**

1-125 ترحم⭘ 2- 125 تشويق⭘ 3-125 ناديده گرفتن(بی اعتنا به شما عبور می کنند)⭘ 4-125 عدم درک شرايط يک فرد نابينا ⭘ 5-125 قطع ارتباط ⭘ 6-125 رفتار کودکانه (مثل يک کودک با شما رفتار می کنند)⭘ 7-125 مثل متکدی ⭘ 8-125 دوستانه⭘

9-125 تمسخر⭘ 10-125 عذرخواهی⭘

در صورت تمايل جانباز: 🡨 126- تلفن تماس جانباز: - 127- همراه:

**128- نام**: ⬜⬜⬜⬜⬜⬜⬜⬜⬜⬜  **129- نام خانوادگی** : ⬜⬜⬜⬜⬜⬜⬜⬜⬜⬜⬜⬜⬜

130- نام و نام خانوادگی تکميل کننده : 131- تلفن تماس تکميل کننده: -
