# Supplementary material for: Quality of life in the Iranian Blind War Survivors in 2007: a cross-sectional study
Source: BMC Int Health Hum Rights. 2010 Aug 21;10:21. doi: 10.1186/1472-698X-10-21 (PMC2936407; doi:10.1186/1472-698X-10-21)
Supplement: Additional file 2 — Blind war survivors' health related quality of life. The file contains standard SF36 Persian version [file 1472-698X-10-21-S2.DOCX]

**دستور العمل: اين پرسشنامه شما را در مورد سلامتي خودتان بررسي مي كند. اين اطلاعات كمك مي كند تا بتوان به ثبت احساسات شما و اينكه شما تا چه حدي توانايي انجام كارهاي روزانه خود را داريد، اقدام كرد.**

**به هر سؤال به همان شكلي كه توضيح داده شده است، پاسخ دهيد. اگر مطمئن نيستيد كه چگونه به يك سؤال پاسخ دهيد، لطفا" بهترين پاسخ ممكن را انتخاب نماييد.**

**1- بطور كلي، سلامتي خود را چگونه توصيف مي نماييد: ( يكي را مشخص نماييد )**

عالي ⭘^1^ بسيار خوب⭘^2^ خوب⭘^3^  متوسط⭘^4^  بد⭘ ^5^

**2- در مقايسه با سال گذشته بطور كلي سلامت خود را در حال حاضر چگونه ارزيابي مي كنيد. ( يكي را مشخص نماييد )**

بسيار بهتراز سال گذشته است⭘^1^  كمي بهتراز سال گذشته است⭘^2^ تقريبا مشابه سال گذشته است⭘^3^

كمي بدتراز سال گذشته است⭘^4^  بسيار بدتراز سال گذشته است⭘^5^

**3- موارد زير شامل فعاليت هايي است كه شما احتمالا" طي يك روز عادي انجام مي دهيد. آيا وضعيت سلامتي شما در حال حاضر اين فعاليت ها را محدود كرده است؟ اگر چنين است به چه ميزان؟ ( از هر عدد يكي را مشخص نماييد )**

| **فعاليت ها** | بله، بسيار محدود شده  است | بله كمي محدود شده  است | خير، اصلا" محدود نشده  است |
| --- | --- | --- | --- |
| **الف- فعاليت هاي سنگين** مثل دويدن، بلند كردن اجسام سنگين، شركت در ورزش هاي قدرتي | **1** | **2** | **3** |
| **ب – فعاليت هاي متوسط** مثل حركت دادن يك ميز، جابجايي جاروبرقي، انجام ورزش هاي سبك | **1** | **2** | **3** |
| **ج**- بلند كردن يا حمل خواربار منزل | **1** | **2** | **3** |
| **د**-بالا رفتن از **چند** راه پله | **1** | **2** | **3** |
| **ه** – بالا رفتن از **يك** راه پله | **1** | **2** | **3** |
| **و**- دولا شدن، زانو زدن يا خم شدن | **1** | **2** | **3** |
| **ز**- راه رفتن براي **بيش از يك كيلومتر** | **1** | **2** | **3** |
| **ح**- راه رفتن براي **بيش از چند كوچه** | **1** | **2** | **3** |
| **ط**- راه رفتن براي **بيش از يك كوچه** | **1** | **2** | **3** |
| **ي**- حمام كردن يا پوشيدن لباس | **1** | **2** | **3** |

1. **آيا طي 4 هفته گذشته** در كار ويا ساير فعاليت هاي روزمره**، به علت وضعيت سلامت جسماني** خود يكي از مشكلات زير را داشته‌ايد**؟ ( از هر رديف يك عدد را مشخص نماييد )**

|  | **بله** | **خير** |
| --- | --- | --- |
| **الف – كاهش مدت زماني** كه صرف كار يا ساير فعاليت ها نموده ايد | **1** | **2** |
| **ب – به كمتر** از آنچه كه تمايل داشته ايد، دست يافته ايد | **1** | **2** |
| **ج-** در انجام كارهايي **خاص** يا ساير فعاليت ها محدوديت داشته ايد | **1** | **2** |
| **د-** در انجام كار يا ساير فعاليت ها دچار **مشكل** شده ايد( مثلا" نيازمند تلاش بيشتري بوده ايد ) | **1** | **2** |

**به صفحه بعد مراجعه كنيد**

1. **آيا طي 4 هفته گذشته در كار و يا ساير فعاليت هاي روزمره، به علت مشكلات روحي خود يكي از مشكلات زير را داشته‌ايد؟**

**( از هر رديف يك عدد را مشخص نماييد )**

|  | **بله** | **خير** |
| --- | --- | --- |
| **الف – كاهش مدت زماني كه صرف كار يا ساير فعاليت ها نموده ايد** | **1** | **2** |
| **ب – به كمتر از آنچه كه تمايل داشته ايد، دست يافته ايد** | **1** | **2** |
| **ج- در انجام كارهايي خاص يا ساير فعاليت ها محدوديت داشته ايد** | **1** | **2** |

1. **طي 4 هفته گذشته سلامت جسماني يا مشكلات روحي شما تا چه حدي فعاليت هاي معمول اجتماعي شما را در رابطه با خانواده، دوستان، همسايگان با مردم مختل كرده بود؟ ( يكي را مشخص نماييد )**

اصلا"⭘^1^ بسيار كم⭘^2^ كمي⭘^3^ تا حدي⭘^4^ زياد⭘^5^ خيلي زياد⭘^6^

**7- طي 4 هفته گذشته چقدر درد داشته ايد؟ ( يكي را مشخص نماييد )**

اصلا"⭘^1^ بسيار كم⭘^2^ كمي⭘^3^ تا حدي⭘^4^ زياد⭘^5^ خيلي زياد⭘^6^

**8- طي 4 هفته گذشته درد تا چه حد در كار معمولي و هميشگي شما اختلال ايجاد كرده بود(هم كار خارج از منزل و هم كار منزل)؟ ( يكي را مشخص نماييد )**

اصلا"⭘^1^ بسيار كم⭘^2^ كمي⭘^3^ تا حدي⭘^4^ زياد⭘^5^ خيلي زياد⭘^6^

**9- اين پرسش ها مربوط به احساسات و وضعيت شما طي 4 هفته گذشته است. لطفا" براي هر سؤال نزديكترين پاسخ به احساس خود را انتخاب كنيد، چه مدتي طي 4 هفته گذشته: ( از هر رديف يك عدد را مشخص نماييد )**

|  | تمام اوقات | اغلب اوقات | خيلي وقت ها | بعضي وقت ها | به ندرت | هيچ وقت |
| --- | --- | --- | --- | --- | --- | --- |
| **الف- فردي فعال بوده ايد؟** | **1** | **2** | **3** | **4** | **5** | **6** |
| **ب- فردي بسيار عصبي بوده ايد؟** | **1** | **2** | **3** | **4** | **5** | **6** |
| **ج- به حدي غمگين بوده ايد كه هيچ چيزي شما را شاد نمي كرده است؟** | **1** | **2** | **3** | **4** | **5** | **6** |
| **د- احساس آرامش و امنيت داشته ايد؟** | **1** | **2** | **3** | **4** | **5** | **6** |
| **ه- خود را پر از انرژي احساس مي كرده ايد؟** | **1** | **2** | **3** | **4** | **5** | **6** |
| **و- خود را غمگين و افسرده احساس مي كرده ايد؟** | **1** | **2** | **3** | **4** | **5** | **6** |
| **ز- احساس ضعف بيش از حد مي كرده ايد؟** | **1** | **2** | **3** | **4** | **5** | **6** |
| **ح- فردي شاد بوده ايد؟** | **1** | **2** | **3** | **4** | **5** | **6** |
| **ط- احساس خستگي مي كرده ايد؟** | **1** | **2** | **3** | **4** | **5** | **6** |

**به صفحه بعد مراجعه كنيد**

**10- طي 4 هفته گذشته، وضعيت جسماني يا مشكلات روحي شما چه مدتي فعاليت هاي اجتماعي شما مختل كرده بود(مثل ديدار دوستان، بستگان وغيره)- ( يكي را مشخص نماييد )**

تمام اوقات ⭘^1^  بيشتر اوقات⭘^2^ بعضي اوقات⭘^3^ بندرت⭘^4^ هيچ وقت⭘^5^

**11- هر كدام از عبارت زير تا چه حدي در مورد شما درست يا نادرست است؟ ( از هر رديف يك عدد را مشخص نماييد )**

|  | كاملا"درست است | تا حدود زيادي درست است | نمي دانم | تا حدود زيادي نادرست است | كاملا"  نادرست است |
| --- | --- | --- | --- | --- | --- |
| **الف- به نظر مي رسد كه من نسبت به ديگر افراد راحت تر مبتلا به بيماري مي شوم** | **1** | **2** | **3** | **4** | **5** |
| **ب- سلامتي من مثل ديگر افرادي است كه مي شناسم** | **1** | **2** | **3** | **4** | **5** |
| **ج- انتظار دارم كه وضع سلامتي ام بدتر شود** | **1** | **2** | **3** | **4** | **5** |
| **د- وضعيت سلامتي من عالي است** | **1** | **2** | **3** | **4** | **5** |

1. تمامي حق و حقوق براي Medical Outcome Trust محفوظ است.
2. 2- استفاده از اين نسخه براي پزو هش هاي علمي در ايران منوط به اجازه كتبي از پزوهشكده علوم بهداشتي جهاد دانشگاهي است.

**نام و نام خانوادگی تکمیل کننده : تاریخ تکمیل فرم : امضاء :**
