# Supplementary material for: Quality of life in the Iranian Blind War Survivors in 2007: a cross-sectional study
Source: BMC Int Health Hum Rights. 2010 Aug 21;10:21. doi: 10.1186/1472-698X-10-21 (PMC2936407; doi:10.1186/1472-698X-10-21)
Supplement: Additional file 4 — General Physical Exam. The file contains history and physical exam of the blind war survivors. [file 1472-698X-10-21-S4.DOC]

**ID Code:** ⬜⬜⬜⬜⬜⬜⬜⬜⬜⬜ اProvince:. . . . . . . . . . . . . . . . . . . . . . . . . .

Name: ⬜⬜⬜⬜⬜⬜⬜ Family: ⬜⬜⬜⬜⬜⬜⬜⬜⬜⬜

**History:**

1. **Personal**
2. **Skin:** 1-1Hair loss *No⭘^1^ Yes⭘^2^* 1-2 Itching *No⭘^1^ Yes⭘^2^* 1-3 Burn *No⭘^1^ Yes⭘^2^*
3. **Mustard gas Exposure** *NO⭘^1^ Yes⭘^2^*
4. **Habits:** 3-1 Gobbler *NO⭘^1^ Yes⭘^2^* 3-2 Cig. Smoking *NO⭘^1^ Yes⭘ ^2^* . . . . . . . . .3-3 Pack Year

3-4 Substance abuse *NO⭘^1^ Yes⭘^2^*

1. **Eyes:** 4-1 pain *NO⭘^1^ Yes⭘^2^* 4-2 Secretion *NO⭘^1^ Yes⭘^2^* 4-3 Irritation *NO⭘^1^ Yes⭘^2^*
2. **Ears:** 5-1 Tinnitus *NO⭘^1^ Yes⭘^2^* 5-2 Secretion *NO⭘^1^ Yes⭘^2^*

5-3 Hearing Loss *NO⭘^1^ Yes⭘^2^*

5-3-1 Unilateral *NO⭘^1^ Yes⭘^2^* 5-3-2 Bilateral *NO⭘^1^ Yes⭘^2^*

1. **Injuries::** 6-1 Accident *NO⭘^1^ Yes⭘^2^* 6-2 Falls: *NO⭘^1^ Yes⭘^2^*  6-1-1- Times:. . . .

6-3 Fractures *NO⭘^1^ Yes⭘^2^*

1. **Head injuries:** *NO⭘^1^ Yes⭘^2^* 7-1 Unconsciousness *NO⭘^1^ Yes⭘^2^*
2. **Breating:** 8-1 Dyspnea *NO⭘^1^ Yes⭘^2^* 8-2 Chronic Cough *NO⭘^1^ Yes⭘^2^*

8-3 Asthma *NO⭘^1^ Yes⭘^2^*

1. **Cardiovascular:** 9-1 Faint: NO⭘^1^ Yes⭘^2^ 9-2 Hypertension NO⭘^1^ Yes⭘^2^

9-3 Hypotension *NO⭘^1^ Yes⭘^2^* 9-4 Cardiovascular attack *NO⭘^1^ Yes⭘^2^*

9-5 Arrhythmia NO⭘^1^ Yes⭘^2^

1. **GI:** 10-1 Appetite Loss *NO⭘^1^ Yes⭘^2^* 10-2 Burping: *NO⭘^1^ Yes⭘^2^* 10-3 Vomiting *NO⭘^1^ Yes⭘^2^*10-4 Constipation NO⭘^1^ Yes⭘^2^ 10-5 Gastritis NO⭘^1^ Yes⭘^2^ 10-6 Diabetes NO⭘^1^ Yes⭘^2^
2. **Neurologic:**11-1 Head Ache *NO⭘^1^ Yes⭘^2^* 11-2 Vertigo *NO⭘^1^ Yes⭘^2^* 11-3 Seizure: *NO⭘^1^ Yes⭘^2^* 11-4 Amnesia *NO⭘^1^ Yes⭘^2^* 11-5 Confusion *NO⭘^1^ Yes⭘^2^*

11-6 Psychological Problems *NO⭘^1^ Yes⭘^2^*

1. **Urinary Tract:** 12-1 Polyuria *NO⭘^1^ Yes⭘^2^*  12-2 Dysuria *NO⭘^1^ Yes⭘^2^*

12-3 hematuria *NO⭘^1^ Yes⭘^2^* 12-4 Incontinency *NO⭘^1^ Yes⭘^2^*

1. **Sexual:** 13-1 Premature Ejaculation: *NO⭘^1^ Yes⭘^2^* 13-2 Erectile Dysfunction *NO⭘^1^ Yes⭘^2^*

13-3 Hyper *No⭘^1^ yes⭘^2^* 13-4 Hypo *No⭘^1^ yes⭘^2^*

1. **Cancer:** *NO⭘^1^ Yes⭘^2^* 14-1  **. . . . . . . . . . . . . . . . . . . . . . . . . . . . . . . . . . . . . . . . . . . . . . . . . . . . . . . . . . . . . . . . . . . . . . . . .**
2. **Drugs:**  *NO⭘^1^ Yes⭘^2^*

15-1**. . . . . . . . . . . . . . . . . . . . . . . . . . . . . . . . . . . . . . . . . . . .** 15-2**. . . . . . . . . . . . . . . . . . . . . . . . . .**

15-3  **. . . . . . . . . . . . . . . . . . . . . . . . . . . . . . . . . . . . . . . . . . . .**  15-4 **. . . . . . . . . . . . . . . . . . . . . . . . . . .**

1. **Others . . . . . . . . . . . . . . . . . . . . . . . . . . . . . . . . . . . . . . . . . . . . . . . . . . . . . . . . . . . . . . . . . . . . . . . . . . . . . . . . . . . . . . . . . . . . . . . . . . . . . . . . . . . . . . . . . . . . . . . . . . . . . . . . . . . . . . . . . . . . . . . . . . . . . . . . . . . . . . . . . . . . . . . . . . . . . . . . . . . . . . . . . . . . . . . . . . . . . . . . . . . . . . . . . . . . . . . . . . . . . . . . . . . . . . . .**
2. **Family**
3. **Cardio Vascular:**  17-1 HTN *NO⭘^1^ Yes⭘^2^* 17-2 Cardiovascular Dis. *NO⭘^1^ Yes⭘^2^*
4. **DM:**  *NO⭘^1^ Yes⭘^2^* 18-1 **. . . . . . . . . . . . . . . . . . . . . . . . . . . . . . . . . . . . . . . . . . . . . . . . . . . . . . . . . . . . . . . . . . . . . . . . . . . . . . .**
5. **Cancer:**  *NO⭘^1^ Yes⭘^2^* 19-2 **. . . . . . . . . . . . . . . . . . . . . . . . . . . . . . . . . . . . . . . . . . . . . . . . . . . . . . . . . . . . . . . . . . . . . . . . . . . . . . . .**
6. **Others: . . . . . . . . . . . . . . . . . . . . . . . . . . . . . . . . . . . . . . . . . . . . . . . . . . . . . . . . . . . . . . . . . . . . . . . . . . . . . . . . . . . . . . . . . . . . . . . . . . . . . . . . . . . . . .**
7. **Physical Exam**
8. **General Appearance:**  21-1Fatty:  *NO⭘^1^ Yes⭘^2^* 21-2 Tiny: *NO⭘^1^ Yes⭘^2^*

21-3 Well:*NO⭘^1^ Yes⭘^2^* 21-4 Pale: *NO⭘^1^ Yes⭘^2^* 21-5 Ill: *NO⭘^1^ Yes⭘^2^*

1. **Vital signs::**22-1 Systolic BP (Supine) / mmHg 22-2 Diastolic BP(Supine) / mmHg

22-3 Systolic BP (Sitting) / mmHg 22-4 Diastolic BP (Sitting) / mmHg

22-5 HR: 22-6 RR:

1. **Height** ⬜⬜⬜ cm **24- Weight** ccc kg **25 Wrist** cc cm
2. **Skin and hair: . . . . . . . . . . . . . . . . . . . . . . . . . . . . . . . . . . . . . . . . . . . . . . . . . . . . . . . . . . . . . . . . . . . . . . . . . . . . . . . . . . . . . . . . . . . . . . . . . . . . . . . . . . . . . . . . . . . . . . . . . . . . . . . . . . . . . . . . . . . . . . . . . . . . . . . . . . . . . . . . . . . . . . . . . . . . . . . . . . . . . . . . . . . . . . . . . . . . . . . . . . . . . . . . . . . . . . . . . . .**

**Thyroid . . . . . . . . . . . . . . . . . . . . . . . . . . . . . . . . . . . . . . . . . . . . . . . . . . . . . . . . . . . . . . . . . . . . . . . . . . . . . . . . . . . . . . . . . . . . . . . . . . . . . . . . . . . .**

1. **Heart**

25-1 Normal S1 no⭘^1^ yes⭘^2^ 25-2 Normal S2 no⭘^1^ yes⭘^2^

25-3 Splitting S1 no⭘^1^ yes⭘^2^ 25-4 Splitting S2 no⭘^1^ yes⭘^2^

25-5 Systolic Murmur no⭘^1^ yes⭘^2^ 25-6 Diastolic Murmur no⭘^1^ yes⭘^2^

1. **Lungs**

26-1Harsh Sounds No⭘^1^ yes⭘^2^ 26-2 Ralls No⭘^1^ yes⭘^2^ 26-3 Ronchi No⭘^1^ yes⭘^2^

26-4 Wheezeno⭘^1^ yes⭘^2^

1. **Abdomen**

27-1 Distended No⭘^1^ yes⭘^2^ 27-2 fatty No⭘^1^ yes⭘^2^ 27-3 Ascieticno⭘^1^ yes⭘^2^

27-4 Scaphoidno⭘^1^ yes⭘^2^

*Palpation:* 27-5 Tenderno⭘^1^ yes⭘^2^ 27-5 Hepathomegalyno⭘^1^ yes⭘^2^

27-6 Splenomegalyno⭘^1^ yes⭘^2^

1. **Lab Findings:**

28-1CBC: **Normal⭘ Abnormal⭘ ↓**

. . . . . . . . . . . . . . . . . . . . . . . . . . . . . . . . . . . . . . . . .

28-2 Blood Sugar: . . . . . . . . . 28-3 BUN: . . . . . 28-4 Cr.:. . . . . . 28-5 Na: . . . . . 28-6 K:. . . . .

28-7 TG: **Normal⭘ Abnormal⭘ ↓** 28-8 Chol.: **Normal⭘ Abnormal⭘ ↓**

. . . . . . . . . . . . . . . . . . . . . . . .

28-9 LDL: **Normal⭘ Abnormal⭘ ↓**  28-10 HDL: **Normal⭘ Abnormal⭘ ↓**

. . . . . . . . . . . . . . . . . . . . . . . .

28-11 LFT: . . . . . . . . . . . . . . . . . . . . . . . . . . . . . . . . . . . . . . . . . . . . . . . . . . . . . . . . . . . . . . . . . . . . . . . ..

1. **Impression: . . . . . . . . . . . . . . . . . . . . . . . . . . . . . . . . . . . . . . . . . . . . . . . . . . . . . . . . . . . . . . . . . . . . . . . . . . . . . . . . . . . . . . . . . . . . . . . . . . . . . . . . . . . . . . . . . . . . . . . . . . . . . . . . . . . . . . . . . . . . . . . . . . . . . . . . . . . . . . . . . . . . . . . . . . . . . . . . . . . . . . . . . . . . . . . . . . . . . . . . . . . . . . . . . . . .**
2. **Plan: . . . . . . . . . . . . . . . . . . . . . . . . . . . . . . . . . . . . . . . . . . . . . . . . . . . . . . . . . . . . . . . . . . . . . . . . . . . . . . . . . . . . . . . . . . . . . . . . . . . . . . . . . . . . . . . . . . . . . . . . . . . . . . . . . . . . . . . . . . . . . . . . . . . . . . . . . . . . . . . . . . . . . . . . . . . . . . . . . . . . . . . . . . . . . . . . . . . . . . . . . . . . . . . . . . . . . . . . . . .**
3. **Physician Name and Signature**
